# The Histopathology of the Appendix in Children at Interval Appendectomy

**DOI:** 10.3390/children8090811

**Published:** 2021-09-16

**Authors:** Federica Pederiva, Rossana Bussani, Vennus Shafiei, Daniela Codrich, Edoardo Guida, Jurgen Schleef

**Affiliations:** 1 Pediatric Surgery, Institute for Maternal and Child Health—IRCCS “Burlo Garofolo”, 34137 Trieste, Italy; daniela.codrich@burlo.trieste.it (D.C.); edoardo.guida@burlo.trieste.it (E.G.); jurgen.schleef@burlo.trieste.it (J.S.); 2 Department of Pathology, Azienda Sanitaria Universitaria Integrata di Trieste, 34137 Trieste, Italy; rossana.bussani@asuits.sanita.fvg.it (R.B.); shafiei.vennus@spes.uniud.it (V.S.)

**Keywords:** interval appendectomy, complicated appendicitis, appendiceal abscess, histopathology

## Abstract

Whilst most surgeons agree that conservative treatment of appendiceal abscess in children is an adequate treatment, the need for subsequent interval appendectomy is still controversial. We analyzed the histopathology in interval appendectomy in search of signs of inflammation. All patients admitted between 2010 and 2017 with appendiceal abscess and scheduled for interval appendectomy were reviewed. The specimens were evaluated for grade of inflammation, type and distribution of cellular infiltrate, presence of necrosis or hemorrhage and infiltrate in the serosa. Forty-two patients had appendiceal abscess and were treated conservatively. Seven underwent emergent appendectomy. Thirty-three out of 35 patients underwent elective interval appendectomy. Thirty-two specimens were revised. Carcinoid tumor or other malignant lesions were not found. All of them presented some amount of inflammation, grade 1 to 2 in 53%, grade 3 to 4 in 47%. Twenty-five percent of the specimens had signs of necrosis accompanied by hemorrhage and in more than the half (53%) the infiltrate extended to the serosa. Conclusions: Although the appendix was mostly found not macroscopically inflamed intraoperatively, histology confirmed a certain grade of inflammation even months after the conservative treatment. No correlation was found between histopathologic findings and lapse of time between abscess treatment and interval appendectomy.

## 1. Introduction

The presence of an appendiceal mass at the time of the diagnosis complicates two to nine percent of the appendicitis [1,2]. It can be suspected in patients with a palpable mass or with more than three days of symptoms, and it is more common in children younger than five years. Immediate surgical treatment of enclosed appendiceal inflammation is associated with a more than three-fold increase in morbidity compared with non-operative management [1]. Therefore, conservative treatment of appendiceal abscess and subsequent interval appendectomy has become an increasingly common practice. The success of this protocol, in terms of decreased complications and hospital stay length compared with traditional immediate operation, has already been demonstrated [3]. However, the risk of recurrent appendicitis as well as the rationale and timing for an interval appendectomy is still under debate. Some authors have suggested that delayed appendectomy is not necessary unless the patients present with recurrent symptoms [1,4]. The risk of recurrence, which tended to happen in the majority of cases within 6 months after the initial hospital stay, had been estimated for 10–14% [1].

The histopathology data on interval appendectomy in children have been scant. We reviewed the pathologic specimens from interval appendectomy in search of signs of inflammation.

## 2. Materials and Methods

After approval by the Institutional Research Board, all patients admitted to our department between 2010 and 2017 with a diagnosis of appendiceal abscess who later underwent interval appendectomy were retrospectively reviewed. The diagnosis was confirmed by the finding of an appendiceal mass at US scan. As previously reported [5,6], children presenting with an appendiceal mass at our centre were managed conservatively with parenteral broad spectrum antibiotic therapy, until the resolution of illness and normalization of inflammatory markers, and with interval appendectomy scheduled electively at least 6–8 weeks after hospital discharge. The standard antibiotic therapy included Ampicillin/Sulbactam (50 mg/kg/dose) every 8 h, Metronidazole (7.5 mg/kg/dose) every 8 h and Tobramycin (5 mg/kg/dose) once a day. Any patient failing to respond to the conservative management and not improving within 72 h of starting the antibiotics underwent an emergent appendectomy.

All patients in whom an emergent appendectomy was undertaken either for the failure of conservative treatment or for developing recurrent appendicitis, which led to an early and unplanned hospital readmission, were excluded from the study.

Demographic data, type and length of parenteral/oral antibiotic therapy, number of weeks between hospital discharge and elective interval appendectomy, intraoperative findings, surgical complications and post-operative follow-up were recorded.

All specimens were retrieved. Hematoxylin and eosin-stained slides from all cases containing at least two cross sections (distal and intermediate) and one longitudinal section of the appendix, were reviewed by two Pathologists, blinded to both clinical data and to the original pathological record. Histopathologic features were assessed with special attention given to grade of inflammation, histological features of the cellular infiltrate and its distribution, presence of necrosis or hemorrhage or abscesses and presence of infiltrate in the serosa. The grade of inflammation was considered 0 in absence of inflammatory cells, 1 (mild) in presence of few scattered inflammatory cells, 2 when multiple small clusters of inflammatory cells (10 − 20/HPF × 10) were seen, 3 if multiple clusters of inflammatory cells (20 − 30/HPF × 10) tend to merge, and 4 (severe) if the inflammation was widespread (>30/HPF × 10).

Finally, we tried to correlate the grade of inflammation with the lapse of time between conservative management and interval appendectomy.

### Statistical Analysis

All continuous data were reported as median with range. Binomial data were reported as percentage.

## 3. Results

Forty-two patients (23 female and 19 male) were admitted to our centre with a diagnosis of appendiceal abscess at a median age of 8.3 years (range 2–17.2 years) during the study period. All of them were initially treated with intravenous (iv) fluids, analgesics and antibiotics. The standard antibiotic therapy was first administered to all patients but one, allergic to Ampicillin, in whom Ciprofloxacin was instead used. Two male patients failed to respond to the conservative management and underwent an emergent appendectomy at a median age of 14.2 years (range 11.5–17 years). Five patients (1 female and 4 male) developed acute appendicitis whilst on a waiting list for interval appendectomy and required emergent appendectomy. In the remaining 35 patients, the conservative treatment with iv antibiotics was prolonged for a median of 7.5 days (range 6–13 days) with normalization of the clinical conditions and inflammatory markers. All of them were discharged with a prescription of oral amoxicillin/clavulanic acid for 5–7 days and a planned readmission for elective appendectomy. Thirty-three out of 35 patients underwent elective interval transumbilical laparoscopic assisted appendectomy (TULAA) 12 weeks (median; range 8–24 weeks) after discharge. Two female patients were lost at follow up and one of them was admitted 8 months later with another appendiceal abscess and underwent emergent appendectomy. In 13 out of 33 patients the appendix was retrocoecal and in 18 out of 33 adhesions between appendix and abdominal wall were found. In one case there was a “vanishing” appendix and in two others a stump appendix was found. No complications occurred during surgery and the patients were discharged after 2–3 days of hospitalization. At follow-up in the outpatient clinic 7–10 days after discharge all patients were in good health, with no complications of the umbilical wound.

The pathology revision could be conducted in the specimens of 32 out of 33 patients. In none of the excised appendix specimens were a carcinoid tumor or other malignant lesions found.

**Figure 1:**
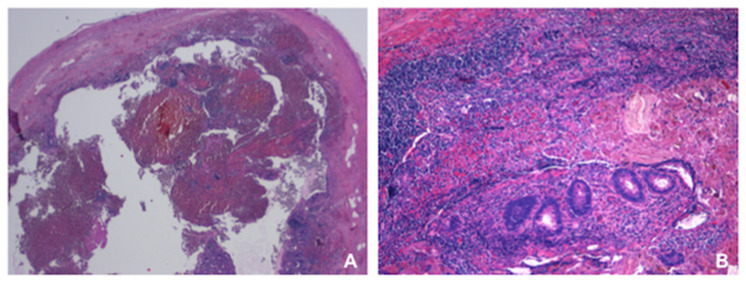
(**A**) Appendix section with grade 4 of inflammation extended also to the serosa and associated with necrosis and hemorrhage (H&E, original magnification ×2.5). (**B**) Appendix section with grade 4 of inflammation and diffuse necrosis (H&E, original magnification ×10).

In all the specimens some amount of inflammation was described (Table 1), grade 1 to 2 in 53% (17/32) of the cases, grade 3 to 4 in 47% (15/32) of the patients. Lymphoid elements were found in all the specimens, mingled with hystiocytes in cases of grade 1 and 2 of inflammation, as sign of chronic inflammation, together with granulomatous elements in appendices with grade 3 and 4 of inflammation, as a result of persistence of acute inflammation or flare of inflammation. In 17 cases eosinophilic infiltration was found. The presence of necrosis was directly related to the grade of inflammation, being more pronounced in grade 3 and 4 (9 out of 15, 60%) (Figure 1B). In 25% of cases necrosis was accompanied by hemorrhage (Figure 1A). A crypt abscess was found only in one case. In 17 out of 32 cases (53%) the infiltrate extended to the serosa (Figure 1A), especially in appendices with grade 3 and 4 inflammation (11/17 cases) (Table 1).

No correlation was found between the histopathologic findings and the lapse of time between abscess treatment and interval appendectomy (Table 2).

## 4. Discussion

Conservative treatment of an appendiceal abscess has the goal to localize the inflammatory process and decrease the risk of complications. Recently, a prospective, randomized trial [7] has demonstrated the superiority of postoperative monotherapy with piperacillin-tazobactam over standard 2-drug therapy with ceftriaxone and metronidazole in preventing development of intra-abdominal abscess in children with perforated appendicitis. Patients with perforated appendicitis treated conservatively were excluded from the study. However, the results of this trial should encourage an extension of the treatment also to the appendiceal abscess treated conservatively.

Nonsurgical treatment could fail in 8–13.6% of the cases [1,8]. In our setting it failed in 7 patients (16.6%), 2 at initial conservative treatment and 5 while waiting for interval appendectomy, resulting in all cases in an urgent appendectomy.

Whilst the conservative management of an appendiceal abscess has long been accepted by most surgeons, the need for interval appendectomy after a successful nonsurgical treatment, however, has been questioned, because the risk of recurrence seems relatively small and the possibility to find a carcinoid tumor or malignancy seems even rarer [1,2,9]. Ein et al. [10] described 10% (one patient) of recurrence among ten cases of appendiceal abscesses treated conservatively and followed up for a range of 6 months to 13 years. It should be noted that three patients required a drainage and probably should not have been included among the group treated conservatively. This should raise the discussion if the patient treated with a drainage procedure should qualified or not for conservative treatment group. The percentage of recurrence increased to 42% in a later study of the same group [11], especially if an appendicolith was associated. Puapong et al. [12] reported a recurrence rate of 8%. In a study conducted by Fawkner–Corbett et al. [2], 12% of the children developed acute appendicitis requiring emergent appendectomy, whilst on waiting list for interval appendectomy. In Svensson et al. report [13], 7 out of 89 patients (almost 8%) had either recurrence of abscess (5) or acute appendicitis (2) after conservative treatment of appendiceal abscess. More recently, Tanaka et al. [14] described 6.2% of recurrence in patients waiting for interval appendectomy and 34.2% in an average 3.4 years of follow up among patients treated conservatively for appendiceal abscess and who chose not to have an interval appendectomy. In CHINA study [15], the recurrence of histologically proven appendicitis among children on active observation after conservative treatment for appendix mass was 12%.

We experienced a recurrence rate of 12% (5 acute appendicitis). In one case, the parents did not agree to the scheduled interval appendectomy, and the child was lost at follow up. Eight months later she presented with a recurrent abscess. Should the scheduled interval appendectomy have been performed, this, perhaps, would not have happened.

The high percentage of recurrence, which raised to 42% when the follow-up was extended to 2 years and 34% in 3.4 years of follow-up, supported the recommendation to perform interval appendectomy in patients with appendiceal abscess treated conservatively. On the other hand, the low rate of complications in elective interval appendectomy [11,16], compared with the high risk of recurrence and re-hospitalization, confirmed the safety of the surgical procedure, and added more support to its appropriateness.

It is generally accepted that the risk of recurrence is highest in the first 6–12 months after the successful conservative treatment of an appendiceal abscess, with more than 80% of recurrences happening within 6 months [10,12,13,17]. For this reason, the surgeons in favor of interval appendectomy recommend performing the procedure between 6 weeks and 3 months [10].

We examined the histopathology of the appendix at the time of interval appendectomy in search of signs of inflammation and we tried to correlate these data with the lapse of time between conservative management of the abscess and interval appendectomy. Some authors have examined the macroscopic appearance and histopathology of interval appendectomy specimens, both in children and in the adult population, finding either no signs of previous inflammation [4,17], or subacute [18] or acute [17,18] or chronic inflammation [2,17,19], or fibrosis [2,18,19], or granulomatous inflammation [19,20]. In some cases, the appendix was found to be obliterated by fibrosis and scarring [18], leading to the conclusion that interval appendectomy could be omitted, because the recurrence was thought dependent on a persistently patent appendiceal lumen. On the other hand, appendices with patent lumen were found to be histologically normal [20]. Thus, the risk of recurrence did not correlate only with the lumen characteristics.

The histopathology data on appendix at interval appendectomy in children have been scant. To the best of our knowledge only two studies addressed specifically the histopathology of interval appendectomy specimens with contrasting results. In one of them (17 specimens) [20] the authors found most of the appendices histologically normal at the time of interval appendectomy, while in the other (14 specimens) [19] they described granulomatous inflammation.

In all our patients, a certain grade of inflammation of the appendix was found at histology, despite that the appendix was mostly not macroscopically inflamed intraoperatively. In almost half (47%) of the cases it was described as grade 3 or 4 and frequently accompanied by necrosis. In more than half (53%) of the patients, especially when the inflammation was grade 3 or 4, the infiltrate extended to the serosa.

Interestingly, we could not find any correlation between the histopathologic findings and the interval of time between abscess treatment and interval appendectomy. All grades of inflammation were found almost at each time lapse.

The finding of persistent inflammation in all appendices at interval appendectomy supported the recommendation of not omitting the interval appendectomy after the conservative treatment of an appendiceal abscess.

Lately [21], the previous undisputed concept of conservatively treating an appendiceal abscess has been questioned, at least among adult patients. In the laparoscopic era an emergency appendectomy is considered a quick, safe and feasible treatment for appendiceal abscess. If this attempt to shift from delayed appendectomy to emergency appendectomy is also applicable to the pediatric patients, remains to be demonstrated and further qualitative studies are needed to substantiate this management.

This study has some limitations, which should be acknowledged. First, the lag time between the end of conservative treatment and the surgical procedure was quite short in a number of cases. Second, it might be speculated that the presence of inflammation could be a remnant of the previous episode. However, the analysis failed to show a relationship between surgical procedure lag time and the degree of inflammation and seems to rule out the hypothesis of slowly resolving previous damage.

In conclusion, the results of this study confirmed that there is still some grade of inflammation in the appendix even several months after the conclusion of the conservative management of the appendiceal abscess, and that a higher grade of inflammation does not correlate with a shorter time between conservative treatment and interval appendectomy. More data are needed to confirm these results.

WHAT IS KNOWN:

- Conservative treatment of an appendiceal abscess localizes the inflammatory process and decreases the risks of complications.
- Nonsurgical treatment could fail in 8–13.6% of the cases.

WHAT IS NEW:

- The appendix was mostly not macroscopically inflamed intraoperatively.
- There are still signs of inflammation in appendix several months after the conclusion of conservative management of appendiceal abscess.
- No correlation was found between the histopathologic findings and the interval of time between abscess treatment and interval appendectomy.

## Figures and Tables

**Table 1:** Histopathologic features of appendices at the time of interval appendectomy.

Grade of Inflammation	*n* %	Necrosis *n*; %	Hemorrhage *n*; %	Necrosis + Hemorrhage *n*; %	Abscesses *n*; %	Infiltrate in Serosa *n*; %
0	-	-	-	-	-	-
1	5 15.6%	-	2 6.2%	-	-	-
2	12 37.5%	1 3.1%	2 6.2%	3 9.3%	-	6 18.7%
3	7 21.8%	2 6.2%	-	4 12.5%	1 3.1%	5 15.6%
4	8 25%	7 21.8%	-	1 3.1%	-	6 18.7%
Tot	32 100%	10 31.2%	4 12.5%	8 25%	1 3.1%	17 53%

**Table 2:** Grade of inflammation of appendices and time lapse before interval appendectomy.

Grade of Inflammation	*n*	8 Weeks	12 Weeks	16 Weeks	24 Weeks
1	5	2	2		1
2	12	6	5	1	
3	7	3	4		
4	8	3	4	1	
Tot	32	14	15	2	1

## Data Availability

The data are available in the results section of the manuscript.

## Abbreviations

HPF	high-power field
kg	kilogram
mg	milligram
TULAA	transumbilical laparoscopic assisted appendectomy

## References

[1] Andersson R.E., Petzold M.G. (2007). Nonsurgical Treatment of Appendiceal Abscess or Phlegmon. Ann. Surg..

[2] Fawkner-Corbett D., Jawaid W.B., McPartland J., Losty P.D. (2014). Interval appendectomy in children clinical outcomes, financial costs and patient benefits. Pediatr. Surg. Int..

[3] Bufo A.J., Shah R.S., Li M.H., Cyr N.A., Hollabaugh R.S., Hixson S.D., Schropp K.P., Lasater O.E., Joyner R.E., Lobe T.E. (1998). Interval Appendectomy for Perforated Appendicitis in Children. J. Laparoendosc. Adv. Surg. Tech..

[4] Willemsen P., Hoorntje L.E., Eddes E.-H.H., Ploeg R.J. (2002). The Need for Interval Appendectomy after Resolution of an Appendiceal Mass Questioned. Dig. Surg..

[5] Codrich D., Scarpa M.G., Lembo M.A., Pederiva F., Olenik D., Gobbo F., Giannotta A., Cherti S., Schleef J. (2013). Transumbilical Laparo-Assisted Appendectomy: A Safe Operation for the Whole Spectrum of Appendicitis in Children—A Single-Centre Experience. Minim. Invasive Surg..

[6] Guida E., Pederiva F., Di Grazia M., Codrich D., Lembo M.A., Scarpa M.G., Rigamonti W. (2015). Perforated appendix with abscess: Immediate or interval appendectomy? Some examples to explain our choice. Int. J. Surg. Case Rep..

[7] Lee J., Garvey E.M., Bundrant N., Hargis-Villanueva A., Kang P., Osuchukwu O., Dekonenko C., Svetanoff W.J., Peter S.D.S., Padilla B. (2021). IMPPACT (Intravenous Monotherapy for Postoperative Perforated Appendicitis in Children Trial). Ann. Surg..

[8] Zhang H.-L., Bai Y.-Z., Zhou X., Wang W.-L. (2013). Nonoperative Management of Appendiceal Phlegmon or Abscess with an Appendicolith in Children. J. Gastrointest. Surg..

[9] Karaca I., Altıntoprak Z., Karkıner A., Temir G., Mir E. (2001). The Management of Appendiceal Mass in Children: Is Interval Appendectomy Necessary?. Surg. Today.

[10] Ein S.H., Shandling B. (1996). Is interval appendectomy necessary after rupture of an appendiceal mass?. J. Pediatr. Surg..

[11] Ein S.H., Langer J.C., Daneman A. (2005). Nonoperative management of pediatric ruptured appendix with inflammatory mass or abscess: Presence of an appendicolith predicts recurrent appendicitis. J. Pediatr. Surg..

[12] Puapong D., Lee S.L., Haigh P.I., Kaminski A., Liu I.-L.A., Applebaum H. (2007). Routine interval appendectomy in children is not indicated. J. Pediatr. Surg..

[13] Svensson J.F., Johansson R., Kaiser S., Wester T. (2014). Recurrence of acute appendicitis after non-operative treatment of appendiceal abscess in children: A single-centre experience. Pediatr. Surg. Int..

[14] Tanaka Y., Uchida H., Kawashima H., Fujiogi M., Suzuki K., Takazawa S., Deie K., Amano H., Iwanaka T. (2016). More than one-third of successfully nonoperatively treated patients with complicated appendicitis experienced recurrent appendicitis: Is interval appendectomy necessary?. J. Pediatr. Surg..

[15] Hall N.J., Eaton S., Stanton M.P., Pierro A., Burge D.M. (2017). Active observation versus interval appendicectomy after successful non-operative treatment of an appendix mass in children (CHINA study): An open-label, randomised controlled trial. Lancet Gastroenterol. Hepatol..

[16] Takahashi Y., Obata S., Matsuura T., Kawano Y., Yanagi Y., Yoshimaru K., Izaki T., Taguchi T. (2021). The experiences of interval appendectomy for inflammatory appendiceal mass. Pediatr. Int..

[17] Hall N.J., Jones C.E., Eaton S., Stanton M.P., Burge D.M. (2011). Is interval appendicectomy justified after successful nonoperative treatment of an appendix mass in children? A systematic review. J. Pediatr. Surg..

[18] Janik J.S., Ein S.H., Shandling B., Simpson J.S., Stephens C.A. (1980). Nonsurgical management of appendiceal mass in late presenting children. J. Pediatr. Surg..

[19] Guo G., Greenson J.K. (2003). Histopathology of interval (delayed) appendectomy specimens: Strong association with granulomatous and xanthogranulomatous appendicitis. Am. J. Surg. Pathol..

[20] Mazziotti M., Marley E., Winthrop A., Fitzgerald P., Walton M., Langer J. (1997). Histopathologic analysis of interval appendectomy specimens: Support for the role of interval appendectomy. J. Pediatr. Surg..

[21] Ahmed A., Feroz S.H., Dominic J.L., Muralidharan A., Thirunavukarasu P. (2020). Is Emergency Appendicectomy Better Than Elective Appendicectomy for the Treatment of Appendiceal Phlegmon?: A Review. Cureus.

